# Efficacy of elastodontic devices in overjet and overbite reduction assessed by computer-aid evaluation

**DOI:** 10.1186/s12903-021-01628-7

**Published:** 2021-05-17

**Authors:** Eleonora Ortu, Davide Pietropaoli, Samuele Cova, Maria Chiara Marci, Annalisa Monaco

**Affiliations:** 1 MeSVA Department, Dental Unit, University of L’Aquila, P.Le S. Tommasi, 67100 L’Aquila, Italy; 2 Cova Samuele, DDS, Freelance, 38023 Cles, Trento, Via Tiberio Claudio Italy

**Keywords:** Orthodontic, Elastodontic device, Overjet, Overbite, Malocclusion

## Abstract

**Background:**

This study aimed to verify the efficacy of two elastodontic devices in overjet (OJ) and overbite (OB) reduction during treatment with the Equilibrator Series II (Eptamed) and Occlus-o-Guide (Sweden & Martina) devices.

**Method:**

Sixty patients aged 7–15 years were enrolled in the study, and were divided into test and control groups. The test group included 30 patients (14 males, 16 females; mean age, 10.66 ± 2.12 years) treated with the EQ (Equilibrator) Series II. The control group included 30 patients (15 males, 15 females; mean age, 10.76 ± 2.52 years) treated with the Occlus-o-Guide. The two groups exhibited the same orthodontic features. The orthodontic criteria were: skeletal and dental class II malocclusion (divisions 1 and 2); and the presence of OJ and OB. Evaluation of OJ and OB was performed at two timepoints: T0 (before starting therapy) and T1 (after 1 year).

**Results:**

At T0, OJ and OB were similar for the two groups; however, at T1, both OJ and OB were significantly lower with the Eptamed device compared to the Occlus-o-Guide device (*p* = 0.0019).

**Conclusions:**

Elastodontic devices improve orthodontic outcomes by aiding orthodontic patient management, diagnosis, and treatment planning, reducing the risk relapse acting on the whole organism and the rehabilitation of the tongue.

## Introduction

Dental overjet (OJ) and dental overbite (OB) are two defects that frequently occur in Caucasian patients. OJ is the extent of horizontal (anterior–posterior) overlap of maxillary central incisors over mandibular central incisors [1]. In comparison, OB is the extent of vertical (superior-inferior) overlap of maxillary central incisors over mandibular central incisors. NANHES III (National Health and Nutrition Survey III) showed that OJ is common in the patients affected by second and third skeletal class malocclusion. An OJ greater than or equal to 5 mm is associated to Class II malocclusion. According to Angle, it is evident in 23% of children, 15% of adolescents, and in 13% of adults. Negative OJ is often associated with Class III malocclusion, but is much less frequent. It occurs in about 3% of American children and 5% of adolescents and adults (https://www.cdc.gov/nchs/nhanes/nhanes_products.htm).

On the vertical plane, increasing OB from 0 to an ideal 2 mm is less frequent in adults compared to children. Severe deep bite (overbite ≥ 5 mm) occurs in about 20% of children and 13% of adults, while a severe open bite (negative overbite ≥ 2 mm) occurs in less than 1% of the population. OJ and OB were separated into five categories for the Italian population based on orthodontic features and the ROMA (Risk of Malocclusion Assessment) index (see published tables in [2, 3]). These orthodontic defects can be associated or not associated with skeletal malocclusions, causing aesthetic and parodontal issues to patients, as well as higher exposure to trauma. The aetiology remains uncertain; however, early treatment of mixed dentition is strongly recommended [4]. Today, orthopedic-functional therapy aims to intercept malocclusions as early as possible. Elastodontic appliances are commonly used to accomplish this objective in orthodontics. Orthodontists have access to a wide range of devices that are easy to wear, acting globally on the stomatognathic system by perfectly integrating with the neuromuscular system and requiring fewer patient check-ups [5].

This study aimed to verify the clinical effect in reducing OJ and OB with two of these devices (EQ Series II [Eptamed] versus Occlus-o-Guide [Sweden & Martina]). We compared dental records before treatment and 12 months after treatment. The results are expected to provide guidelines on the most suitable devices for use in practice.

## Materials and methods

### Study sample

This study was carried out in accordance with the fundamental principles of the Declaration of Helsinki. It was approved before commencement by the Ethics Committee of the University of L’Aquila, Italy (no. 16137/2016). One hundred and fifteen patients aged 6–16 years were clinically examined at the Dental Clinic of the University of L’Aquila, Italy. The same clinician performed all examinations. Examinations included the acquisition of dental panoramic radiographs according to European guidelines on radiation protection in dental radiology, extraoral and intraoral photographs, and alginate impressions of both dental arches. Based on these data, the orthodontist created a treatment plan specific to each patient, following the index of orthodontic treatment needs described by Brook and Shaw [6]. The following exclusion criteria were applied: IOTN (INDEX OF ORTHODONTIC TREATMENT NEED) index > 4; presence of epilepsy, systemic disease, TMD, or periodontal disease; and lack of written informed consent from a parent or legal guardian. Inclusion criteria were: skeletal and dental class II malocclusion (divisions 1 and 2); and the presence of OV and OB. Ultimately, 60 patients aged 7–15 years were enrolled in the study, and separated into test and control groups. The test group included 30 patients (14 males, 16 females; mean age, 10.66 ± 2.12 years), who were treated with the EQ Series II. The control group included 30 patients (15 males, 15 females; mean age, 10.76 ± 2.52 years), who were treated with the Occlus-o-Guide. The two groups exhibited the same orthodontic features. OJ and OB were evaluated at two timepoints: T0 (before starting therapy) and T1 (after 1 year). Alginate impression of the dental arches of the two groups were taken by the same orthodontist (AM) at T0 and T1. Dental stone models were constructed using white stone. Plaster models were sent to DTW (Dental Team Work, Preturo, L’Aquila) for scanning and conversion to 3D virtual models using ITero Intraoral scanning. Variables of the study were OJ and OB, which were evaluated using this virtual digital technique.

### Experimental settings

Each patient in the test group received a medium hardness, orange EQ class II device that was suitable for their dentition phase [7]. This device had a similar shape to a mouthguard, and embraced both dental arches, reaching distally to cover the last molars present in the arch. There are several measures, based on the distance between the palatal cusps of the first premolars. The patient fits the upper and lower splints over their teeth. The device is activated by biting, depending on the soft elastic forces generated by muscle energy. The activator is worn overnight. The equilibrator is a type of orthodontic appliance that stimulates growth and, through the input of muscle movements, elicits tissue development toward a suitable chewing function. Biting this elastomeric device balances tension to the sphenobasilar synchondrosis, based on osteopathic medicine and philosophy [7].

Each patient in the control group received a G-type (for mixed dentition) Occlus-o-Guide device, depending on their dentition phase. This devise is constructed from an elastomeric material. It is a preformed activator that is considered ideal for use in the early to late mixed dentition phase. This appliance is available in various sizes. The most appropriate size is chosen by measuring the distance between the distal wall of the upper left lateral incisor and the distal wall of the upper right lateral incisor. It is also possible to measure it using the lower lateral incisors. All measurements are completed using a special ruler provided by the manufacturer. Along with the activator properties of this device, it is ideal for correcting class II malocclusion, because it is based on tooth size. These appliances are called EGAs (Eruptive Guidance Appliances), as they also function as a positioner, correcting overbite, and correcting mild to moderate crowding. This device provides depressive forces to the front teeth, while simultaneously causing the posterior elements to erupt in their optimal vertical position. It is necessary, however, to intervene when they erupt, before the periodontal fibers stabilize the definitive vertical level of posterior elements. It is also a myofunctional regulator that tends to properly rebalance muscle forces. It rehabilitates the posture of the tongue, re-educates atypical swallowing, and stimulates correct breathing. The patients were instructed to use the device overnight [5, 8, 9]. The orthodontist checked patients every 30 days to evaluate eventual modifications for optimize the execution of the device.

## Results

**Fig. 1:**
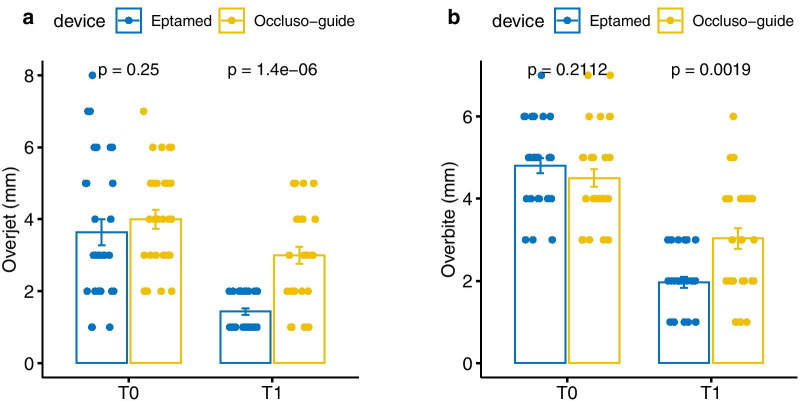
Bar plot of total OJ and OB values stratified by timing according to group "Eptamed" and "Occlusoguide"

The Wilcoxon test was used for within-group comparisons. Statistical significance was set at *p* < 0.05. The results are shown in Fig. 1. Data are presented as mean (standard deviation).

At T0, OJ and OB were similar in both groups. In contrast, at T1, both OJ and OB were significantly lower for the Eptamed device compared to the Occlus-o-Guide device (*p* = 0.0019; Fig. 1).

## Discussion

In addition to achieving the aesthetic goal, orthodontics aim to attain the correct neuromuscular balance of the stomatognathic system at an early stage of growth [5, 10]. To accomplish this, collaborations with specialists from other hospital branches are required, including ENS doctors, physiotherapists, ophthalmologists, and orthopedists. Such collaborations allow the growing patient to attain proper body function [11]. Patients in the second skeletal class malocclusion have a complex pattern of growth. There is a clear correlation between oral breathing and malocclusion; however, published data are conflicting [12]. Both genetic and environmental factors impact an already genetically determined pattern. In a small percentage of patients, the oral-respiratory problems exist related to ENT pathologies [13]. The remaining part develops due to it being more comfortable for the patient to breathe through the mouth. Hence, a series of alterations arise, which are linked to the passage of air through the mouth. These alterations include evident dark circles, difficulty in concentrating, inflammatory pathologies, low posture of the tongue and interposition between the arches (OJ), contraction of transverse diameters, and incorrect posture. All of these factors translate into the entire body suffering.

Here, we demonstrated how elastodontic devices could be used in addition to correcting OJ and OB to improve the general condition of the body. These devices are both simple to use and comfortable. They eliminate functional disorders of the stomatognathic system and act in a three-dimensional way within the oral cavity, improving breathing, swallowing, and postural problems. They are associated with targeted functional exercises, and can be used in association with other orthodontic therapies in progress. Both techniques analyzed here improved the degree of OJ and OB between T0 and T1, even 1-year after the start of therapy. In particular, patients wearing EPTAMED (test group) had a statistically significant improvement in values. Finally, these removable appliances allow the patient to maintain proper oral hygiene at home, even though significant alterations to microbiota tend to be registered 1 month after the start of treatment; however, these devices impact oral bacteria less compared to fixed devices [14–16]. The limitations of this study include the small sample size and relatively short (12-month) orthodontic evaluation period. It would be useful to repeat this study with a larger, and possibly more homogeneous, cohort.

## Conclusions

The results of this study indicate that the use of the elastodontic devices might aid the management, diagnosis, and treatment planning of orthodontic patients, in parallel to improving orthodontic outcomes. In particular, these devices reduce the risk of relapse acting on the whole body of patients, and facilitate the rehabilitation of the tongue.

## Data Availability

The datasets used and analyzed during the current study are available from the corresponding author on reasonable request.

## References

[1] Nguyen QV, Bezemer PD, Habets L, Prahl-Andersen B (1999). A systematic review of the relationship between overjet size and traumatic dental injuries. Eur J Orthod.

[2] Grippaudo C, Paolantonio EG, Pantanali F, Antonini G, Deli R (2014). Early orthodontic treatment: a new index to assess the risk of malocclusion in primary dentition. Eur J Paediatr Dent.

[3] Grippaudo C, Paolantonio EG, Deli R, La Torre G (2008). Orthodontic treatment need in the Italian child population. Eur J Paediatr Dent.

[4] Schatz JP, Ostini E, Hakeberg M, Kiliaridis S (2020). Large overjet as a risk factor of traumatic dental injuries: a prospective longitudinal study. Progress Orthod.

[5] Ortu E, Barrucci G, Aprile G, Guerrini L, Pietropaoli D, Monaco A (2020). Electromyographic evaluation during orthodontic therapy: comparison of two elastodontic devices. J Biol Regul Homeost Agents.

[6] Brook PH, Shaw WC (1989). The development of an index of orthodontic treatment priority. Eur J Orthod.

[7] Aprile G, Ortu E, Cattaneo R, Pietropaoli D, Giannoni M, Monaco A (2017). Orthodontic management by functional activator treatment: a case report. J Med Case Rep.

[8] Farronato G, Giannini L, Galbiati G, Grillo E, Maspero C (2013). Occlus-o-Guide(R) versus Andresen activator appliance: neuromuscular evaluation. Prog Orthod.

[9] Farronato GGE. Dispositivo ortodontico preformato: occlus-o-guide. 2008; https://www.sweden-martina.com/it_it/cases/ortodonzia-1903/casi_clinici-16886/dispositivo_ortodontico_preformato_occlus_o_guide-1944.html.

[10] Ortu E, Pietropaoli D, Marchetti E, Marchili N, Marzo G, Monaco A (2018). Bruxism in children: use of the functional plane of Monaco (FPM). Eur J Paediatr Dent.

[11] Ortu E, Pietropaoli D, Ortu M, Giannoni M, Monaco A (2014). Evaluation of cervical posture following rapid maxillary expansion: a review of literature. Open Dent J.

[12] Grippaudo MM, Quinzi V, Manai A, Paolantonio EG, Valente F, La Torre G (2020). Orthodontic treatment need and timing: assessment of evolutive malocclusion conditions and associated risk factors. Eur J Paediatr Dent.

[13] Souki BQ, Pimenta GB, Souki MQ, Franco LP, Becker HM, Pinto JA (2009). Prevalence of malocclusion among mouth breathing children: do expectations meet reality?. Int J Pediatr Otorhinolaryngol.

[14] Lucchese A, Bondemark L, Marcolina M, Manuelli M (2018). Changes in oral microbiota due to orthodontic appliances: a systematic review. J Oral Microbiol.

[15] Manuelli M (2012). A peaceful man. Progress Orthod.

[16] Pietropaoli D, Del Pinto R, Ferri C, Ortu E, Monaco A (2019). Definition of hypertension-associated oral pathogens in NHANES. J Periodontol.

